# Retraction: Silencing CircHIPK3 sponges miR-93-5p to inhibit the activation of Rac1/PI3K/AKT pathway and improves myocardial infarction-induced cardiac dysfunction

**DOI:** 10.3389/fcvm.2022.1125572

**Published:** 2023-01-04

**Authors:** Frontiers Editorial Office

The journal retracts the 30 April 2021 article cited above.

Following publication, concerns were raised regarding the integrity of the images in the published figures. The authors failed to provide a satisfactory explanation and complete raw dataset during the investigation, which was conducted in accordance with Frontiers' policies. Given the concerns about the validity of the data and the figures, and the lack of complete raw data, the article has been retracted.

This retraction was approved by the Chief Editors of Cardiovascular Medicine and the Chief Executive Editor of Frontiers. The authors agree to this retraction.

